# Globalization, economic development, and corruption: A cross-lagged contingency perspective

**DOI:** 10.1057/s42214-020-00091-5

**Published:** 2021-02-22

**Authors:** Rachida Aïssaoui, Frances Fabian

**Affiliations:** 1 Department of Management, College of Business, Ohio University, Athens, OH 45701 USA; 2 University of Memphis, Memphis, USA

**Keywords:** globalization, economic development, corruption, formal and informal institutions, resource dependence, cross-lagged panel design, longitudinal methods

## Abstract

The 2020 health and economic crisis has exacerbated tensions and debates over whether globalization benefits economic development, as countries face both pressures to enhance economic opportunities through globalization and populist movements seeking protection from global forces. We first review perspectives that offer competing evidence about the role of globalization in regard to economic development and corruption. Drawing on resource dependence and institutional theory, we test the two contingencies of the country’s stage of economic development (low, lower-middle, upper-middle, high) and the globalization dimension (economic, social, political) at play to reconcile competing findings. Using a cross-lagged panel design, we show that these contingencies significantly explain when and what type of globalization can benefit a country’s economy and affect corruption. In doing so, the study provides a platform for future research, and identifies important patterns that can better guide policymaking. Among other results, we find low-income countries’ GDP and corruption benefit the most from the formal dimensions of globalization. With increased wealth, countries are more responsive to the legitimacy accrued with the informal dimensions of globalization, which we find comes at the expense of economic efficiency for high-income countries.

## INTRODUCTION

The 2020 COVID-19 pandemic crisis has highlighted the stark contradiction faced by national policymakers and multinational enterprise (MNE) decision-makers: on the one hand, firms and regulators in countries are revisiting their continued ability to provide essential goods, and accordingly to consider options of greater de-globalization and nationalization (Petricevic & Teece, 2019; Witt, 2019). On the other hand, these same decision-makers are cognizant that the extensive integration of the world economy will demand continued globalization and greater international cooperation to rebuild strength in devastated national markets (Koning, Mertens, & Roosenboom, 2018). Consistent with understandings that globalization trends have been historically nonlinear (Lalountas, Manolas, & Vavouras, 2011), the damaged economic aftermath of the pandemic will represent an important bifurcation point that will require both governments and MNEs to make consequential choices in the near term (Lorenzen, Mudambi, & Schotter, 2020).

Accordingly, policymaking over global integration elicits fundamental uncertainties that lead to great fractiousness. Indeed, research is spotty and mixed regarding the key critiques of, and encomiums to, globalization. While globalization is widely argued to increase a country’s measurable GDP (Bhagwati, 2004), this position is controversial and debated, with theories and evidence pointing to both positive and negative effects (Firebaugh & Goesling, 2004). This circulation of theories with conflicting assumptions and conclusions (e.g., corruption as sand vs. grease in the wheels of commerce), along with the ensuing mixed results from empirical tests, has led political leaders to face formidable obstacles in decision-making (Ugur, 2014) especially in predicting how globalization – defined here as the intensification of economic, political, social, and cultural relations across borders (Holm & Sorensen, 1995: 1) – may uniquely impact their particular country’s economic development. This ambiguity has helped foment widespread political conflict encapsulated in recent populist movements (Hoekman & Nelson, 2018; Ozawa, 2019; Rodrik, 2018). As such, the question “Does globalization increase a country’s wealth?” remains high on the international research agenda (Bryant & Javalgi, 2016; Judge, McNatt, & Xu, 2011; Koning et al., 2018).

In fact, even the advocates of globalization acknowledge that economic growth does not always follow increased integration (Bhagwati, 2004; Krugman, 2008). When they concede this point, though, they often turn to the country’s institutional quality, such as institutions of economic freedom (Bryant & Javalgi, 2016) and governance (Abotsi, 2018), to explain this disappointing result. Among such factors, corruption has been notable as a “powerful force in international business” (Di Pietra & Melis, 2016: 693) in its ability to explain different globalization impacts (Rodrik, 2011). Despite this compelling argument that the globalization–development relationship is contingent upon the “right” institutional context, it has become increasingly evident that what “right” refers to is also highly contested (Aguilera et al., 2008).

Returning to the theoretical causal mechanisms for globalization’s effects – specifically in relation to corruption and economic development – reveals the need to test for contingencies that can drive the significance and direction of relationships. In particular, while our understanding is grounded in economic perspectives, recent decades have increasingly shown that institutions matter (Kim, Kim, & Hoskisson, 2010; Peng et al., 2017). Accordingly, we revisit these relationships by incorporating two major contingencies: a resource dependence contingency of the country stage of economic development (low, lower-middle, upper-middle, and high income), and an institutional contingency in the globalization dimension (economic, social, and political). Doing so allows us not only to move away from the “one-size-fits-all” approach (Aguilera et al., 2008; Donaldson, 2001), but also to depart from the insistence that globalization is principally an economic phenomenon (Lalountas et al., 2011).

Furthermore, we take a longitudinal approach using a cross-lagged panel design as it provides the most powerful evidence for causality. This approach, after considerable incorporation as a mainstay in medical research, has begun to offer compelling insights into organizational research (e.g., Lang et al., 2011). The treatment of globalization data using this approach is a critical contribution here; it responds to skepticism over the reliance on cross-sectional analyses in globalization research (Dimant & Tosato, 2018), and answers continuing calls for more longitudinal methods (Akhter, 2004; Asongu, 2012).

These theoretical and methodological enhancements indicate startling differences across country stages in how different globalization dimensions may impact both future development and levels of corruption in countries. Our results indicate that increasing globalization may be beneficial, negligible, or detrimental to a country’s well-being based on the country’s relative economic standing and the distinctive influence of the globalization dimension. By identifying the significance of these contingencies, we tie our findings to the current globalization debates that are animated by perspectives that differ on both the proposed causal direction and sign of relationships. Policymakers can then move the discussion past incompatible positions toward discovering just when different perspectives are likely to hold, and importantly, why.

We first offer a brief overview of the perspectives on prominent theoretical linkages among globalization, economic development, and corruption. We then justify testing our two contingencies from theory. The methods and analyses are then introduced, with reported results. The research concludes with a discussion of the findings, limitations, and compelling options for future policy and research.

## GLOBALIZATION IMPACTS: DEBATES AND COMPETING PERSPECTIVES

In September of 2016, in the face of stagnating growth in world trade, Roberto Azevedo, Director General of the World Trade Organization (WTO), claimed:

The dramatic slowing of trade growth is serious and should serve as a wake-up call. It is particularly concerning in the context of growing anti-globalization sentiment. We need to make sure that this does not translate into misguided policies that could make the situation much worse, not only from the perspective of trade but also for job creation and economic growth and development, which are so closely linked to an open trading system (WTO, 2016).

This statement reflects widely accepted views from economic theory about the role of globalization in economic development, while simultaneously acknowledging that rampant controverting perspectives endanger this fragile consensus.

The powerful critiques of globalization reflect a complex web of dynamics and interactions that either link globalization to development directly, or condition this relationship to a country’s institutional quality. In addition, the impacts of globalization tend to spawn conflicting discursive frames on the interpretation of these trends (Fiss & Hirsch, 2005), which both reflect and result in basic disputes over the relationships among globalization, (economic) development, and corruption, as summarized in Table 1.

**Table 1:** Overview of arguments on globalization’s impacts and its relationships to development and corruption

Debate	Position	Theory	Example thesis
Globalization’s impacts on development	Increases *KOF → (*+*) GDP*	Wealth	Transnational investments provide resources to increase growth (Habib & Zurawicki, 2002)
	Decreases *KOF → (*-*) GDP*	Impoverishment through exploitation	Exploitation of less-developed economies substitutes for their own internal progress (Carr & Chen, 2001)
		Impoverishment through outsourcing	Outsourcing to lower-paid countries widens income inequality in advanced economies (Krugman, 2008)
Globalization’s impacts on corruption	Reduces *KOF → (*-*) CPI*	Institutional diffusion	Accountability to international partners leads to pressure on the administrative state to combat corruption (Akhter, 2004)
	Increases *KOF → (*+*) CPI*	Institutional contagion	Corruption is contagious, and therefore spreads with globalization (Das & DiRienzo, 2009)
Development’s impacts on corruption	Reduces *GDP → (*-*) CPI*	Compensating mechanism	Because corruption is used to compensate for low wages, increased wealth will reduce the need to resort to corruption (DiRienzo et al., 2007)
	Increases *GDP → (*+*) CPI*	Rent-seeking	Increased wealth opens up new opportunities for rent-seeking (Ades & Di Tella, 1999)
Corruption’s impacts on development/globalization	Reduces *CPI → (*-*) GDP * *CPI → (*-*) KOF*	Sand in the wheels	Corruption acts as a tax that discourages investment and growth (Mauro, 1995), and discourages foreign investment (Rose-Ackerman, 1978)
	Increases *CPI → (*+*) GDP* *CPI → (*+*) KOF*	Grease the wheels	Corruption can facilitate development and globalization by bypassing costly and onerous regulations or compensating for institutional voids (Nye, 1967)

## GLOBALIZATION AND ECONOMIC DEVELOPMENT

### Wealth vs. Impoverishment

The ability for globalization to raise the wealth of nations is perhaps the most pervasive, and well-substantiated, assumption in this research arena (Bhagwati, 2004). The central argument is that globalization supports a nation’s development by spreading industrialization (Firebaugh & Goesling, 2004), and encouraging foreign direct investment (Habib & Zurawicki, 2002).

For the last 20 years, this optimism has been dampened by a realization that globalization does not always meet its promises (Krugman, 2008). Two overarching theories emerged as a result of disappointing results for both developing and developed countries. First is the theory that we refer to as *impoverishment through exploitation*. Its importance notwithstanding, there is little focus in the management literature on the perception –shared by a considerable subset of the anti-globalization movement – that globalization is a force for impoverishing countries (Carr & Chen, 2001), and that this situation is further aggravated by the disproportionate power held by MNEs to influence national policies (Lorenzen et al., 2020).

The notion that globalization may also be detrimental for developed countries has only recently gained traction. In fact, even the most fervent advocates of globalization have revised their position, observing that outsourcing from developed to developing countries has become a serious obstacle to the economic health of developed countries (Krugman, 2008; Rodrik, 2018). Under this perspective, which we refer to as *impoverishment through outsourcing*, globalization displaces the wealth outward from developed countries. This theory has become so popular that many developed countries –the same countries traditionally known to promote globalization as a central policy to support economic growth– are now turning to increased protectionism (see Trump’s trade war with China, or Brexit).

Rodrik (2018) has related populist, anti-globalization, sentiment to these two underlying reasons, noting that our construct of *impoverishment through exploitation* is more likely a tool used by left-wing politicians (e.g., Bernie Sanders as referenced by the author). Alternatively, the perspective of *impoverishment through outsourcing* has become a powerful instrument used by right-wing politicians (Donald Trump was referenced here) as they blame globalization for their countries’ slowing growth and preach protectionism as a response (see also Hoekman & Nelson, 2018; Ozawa, 2019).

## THE ROLE OF CORRUPTION IN THE GLOBALIZATION–DEVELOPMENT RELATIONSHIP 

Challenged to settle the debate over whether globalization enriches or impoverishes economies, scholars have turned to corruption as a possible explanation for competing findings, arguing that, absent corruption, globalization should result in increased development. Corruption, defined as the abuse of entrusted power for private gain (Transparency International, 2017a), is the object of increasing interest given its wide-ranging negative impacts on many domains of modern societies. Yet, although a wide consensus recognizes corruption as a key mechanism affecting the globalization–development relationship, research findings continue to be mixed, thus revealing that there is considerable complexity to this relationship. Specifically, confusion arises as to the direction and sign of these relationships and whether the variables are endogenous or exogenous to the observed country trajectory.

Studies in this area can be summarized under three main debates: (1) Is globalization a vehicle for transparency or does it, conversely, spread corrupt practices? –referred to here as the *institutional diffusion vs. contagion* debate; (2) Is the wealth generated from globalization reducing corruption, or conversely, does it open new opportunities for rent-seeking behaviors? –referred to as *compensating vs. rent*-*seeking* theories; and, lastly, (3) To what extent is corruption facilitative or constraining of development and globalization? –referred to as *sand vs. grease* in the wheels of commerce and globalization.

### Institutional Diffusion vs. Contagion

Firms import and export corporate governance practices in the course of their international business (IB) activities (Koning et al., 2018). The more prevalent side of the debate argues that, along with its touted ability to increase economic growth, globalization can notably combat corruption. Through a process of *institutional diffusion*, “good” practices travel across nations of the world, creating a convergence of these practices (Meyer et al., 1997). Specifically, globalization is said to bring about new accountability mechanisms as international trading partners concerned about corruption pressure the administrative state to lower corruption (Akhter, 2004). Institutional diffusion also results from peer effects driven by membership in supranational organizations (Olabisi, 2019), international mergers and acquisitions (Erez & Gati, 2004), and adoption by neighboring countries and influential organizations (Bahoo, Alon, & Paltrinieri, 2020). Lalountas et al. (2011: 645) nicely summarize this perspective: “Many international institutions consider globalization as a powerful tool to fighting corruption since it presupposes structural and institutional reforms. … These reforms constitute the transmission channel via which globalization affects the control of corruption”.

The opposite argument posits that greater international interaction may be problematic to the extent that corruption is “contagious”. First is the argument that MNEs entering a foreign country with high levels of corruption may face pressures to engage in corrupt practices (Di Pietra & Melis, 2016; Meyer, Li, & Schotter, 2020). Second, globalization can allow home countries to export their own corrupt practices (Das & DiRienzo, 2009), which contradicts findings that emerging market MNEs may very well internationalize to escape the institutional voids in their home markets (Marano, Tashman, & Kostova, 2017). We thus refer to the perspective that globalization fosters corruption as *institutional contagion* to contrast the phenomenon from the beneficial impacts expected from institutional diffusion.

### Compensating vs. Rent-seeking

Research findings that countries with the lowest levels of per-capita economic wealth tend to be the most corrupt (Aidt, Dutta, & Sena, 2008) explain that this is because the proceeds from corrupt practices are used as a means to substitute for undervalued wages (Yenkey, 2015), or to more equitably allocate resources in heavily distorted economies (DiRienzo et al., 2007). Under such a view, corruption acts as a *compensating mechanism,* and will decrease when access to new sources of income opens up with improved economic conditions.

This position is contested, notably for its assumption that wealthier countries consistently move toward less corruption. Instead, evidence abounds that wealthier countries can exhibit high levels of corruption. For instance, Ades and Di Tella (1999) point to oil-rich countries as having the prominent characteristic of generating a substantial portion of government revenues from the oil market alone, a situation that, they argue, benefits government officials with access to this valuable market. More generally, as wealth increases, so do opportunities for rent-seeking behaviors (Escresa & Picci, 2016), a condition we refer to as the *rent*-*seeking* perspective.

### Sand vs. Grease the Wheels

These debates are further complicated by the likelihood that other trajectories may be at work, with research showing that the direction of causality of these relationships may in fact be reversed (Méon & Sekkat, 2005; Yi et al., 2019). Considering that it may be corruption that impacts globalization and/or development, research has commonly characterized corruption as a negative force (Jensen, Li, & Rahman, 2010). This *sand in the wheels* perspective posits that corruption is most strongly associated with dampening a country’s capability to develop (Jensen et al., 2010; Mauro, 1995), essentially by hampering the investment climate and quality (Habib & Zurawicki, 2002). For instance, Rose-Ackerman (1978) noted that organizations such as the World Bank or the International Monetary Fund were reluctant to support investment in corrupt countries. Corruption, under this perspective, also represents an additional tax, and therefore increases transaction costs (Mauro, 1995). Hence, the country’s ability to both globalize and grow is strongly compromised.

However, even this position is controversial with research positing that corruption may also, under certain conditions, act to increase development and globalization, a perspective referred to as *grease the wheels*. Early on, Nye (1967) articulated how corruption allows the disposition of public benefits, for instance by gaining private capital for capital formation, cutting red tape, and providing routes to entrepreneurship. Krammer (2017) similarly found that corruption had a positive effect on start-up business formation in contexts with onerous national-level regulations. Under this view, corruption may act as a type of efficiency-enhancing mechanism in weak institutional environments (Yi et al., 2019).

It is worth noting that the complexity of this debate is further reinforced by recent studies revealing that the proceeds from corruption may be much more hidden, widespread, and difficult to trace than previously thought. Das and DiRienzo (2009: 34) noted that globalization has made the detection of corrupt practices more difficult given the extensive use of electronic commerce and offshore financial centers. There is also broad evidence of decoupled practices by advanced economies actors who, despite their reputation for transparency and social responsibility, are also largely relying on this opaqueness to engage in corrupt practices (Abotsi, 2018; Cuervo-Cazurra, 2018). Recently, the Panama Papers marked a new turn in our appreciation of the extent of this problem: this infamous journalistic event revealed that a wide variety of actors and countries (with very few countries not represented on this list) were engaged in illegal financial flows (Kar & Spanjers, 2015).

Thus, it may be that any support for the grease or sand in the wheels arguments may be contingent upon the type of corruption at play. Corruption consists of two broad types, grand and petty corruption, the first linked to political and economic elites, the second to “street-level” bureaucrats and citizens (Ang, 2020). Given that illicit financial flows are concerned with grand corruption (Kar & Spanjers, 2015), it may just be that conclusions about research investigating whether corruption greases or sands the wheel are more likely ascertaining petty corruption trends rather than grand corruption impacts.

To anchor our investigation on these dynamics, we now revisit some prominent assumptions on how country contexts enact unique conditions that connect globalization with development and corruption. Finding significance to these contingencies in our data can extricate positions from irreconcilability, and better inform the decision-making for globalization and trade policy about likely trends and impacts.

## RESOURCE DEPENDENCE AND INSTITUTIONAL CONTINGENCIES

All of the above theoretical perspectives include findings that imply the relationships are affected by their context. Specifically, our literature review identified two prominent contextual factors that likely bound the relationships of interest. First, the country’s stage of economic development has been advanced as a central contingency, in that it taps the levels of resource dependence that can influence globalization relationships (Brandl, Darendeli, & Mudambi, 2019; Cuervo-Cazurra, Gaur, & Singh, 2019; Rodrik, 1982; Wilson, 2011).

Second, research has recently turned to institutional theory to explain different trajectories of globalization and development, arguing against the common tendency to derive policy prescriptions that “rely on universal notions of best practice” (Aguilera et al., 2008: 475). We thus respond to recent calls to increase attention to the different institutional aspects of globalization to shed additional light on globalization’s impacts on, and relationships to, development and corruption. This approach aligns with the view that globalization is inherently a change process, more specifically a standardization process through which economies, societies and policies tend towards global harmonization (Meyer et al., 1997).

Together, these two contingencies allow us to account for both the initial resource dependence boundaries of these relationships, and the institutional drivers that underlie the observed country trajectories. This approach further responds to Cuervo-Cazurra’s (2016) call to combine different theoretical perspectives in the study of macro-level phenomena such as corruption. He further noted resource-dependency theory in particular helps to understand the causes of corruption, whereas the focus of institutional theory on change processes helps explain its consequences (Cuervo-Cazurra, 2016).

Sherer and Lee (2002) also noted the increased predictive validity from using these two theories in combination. While both theories describe how organizational and national actors face competitive pressures, they differ in their explanations for the responses made to these competitive pressures. Similarly, Hessels and Terjesen (2010: 206) explained that, “While resource-dependency theory argues that dependence on other actors is related to need for resources, institutional theory predicts that organizations are inclined to imitate the behavioral norms of other actors in the organization field”. Finally, considerable evidence indicates that the effectiveness of different kinds of institutions (i.e., formal vs. informal) is contingent upon the country’s stage of development (Brandl et al., 2019; Darendeli & Hill, 2016; Doner & Schneider, 2016).

## RESOURCE DEPENDENCE CONTINGENCY: THE COUNTRY’S STAGE OF ECONOMIC DEVELOPMENT

Resource dependence theory is the study of how the behaviors of organizations and nations are influenced by their need to procure external resources (Pfeffer & Salancik, 1978). As such, a country’s level of development will strongly determine its response to, and influences by, higher-resourced foreign actors. At the firm level, internationalization has been linked to pursuing a strategy to overcome resource scarcities (Hessels and Terjessen, 2010). Resource dependence power asymmetries have also been linked to corruption, as MNEs’ access to certain markets may be contingent upon the provision of bribes (Cuervo-Cazurra, 2016). National-level resource dependencies have also been discussed to understand how countries overcome their resource scarcities through imports (Rodrik, 1982). For instance, Wilson (2011) examines China’s interest in Australia’s iron ore and coal mining sectors, and the Australian government’s response to this new “minerals boom”. Drawing from a resource-dependency model, Yuchtman-Yaar & Inbar (1986) even found resource dependencies influenced the social distances and political conflicts among Arab and Jewish Israeli, Palestinians, and Egyptians. Lastly, supranational organizations such as the International Monetary Fund (IMF) or the World Bank also hold influential power over those governments who rely on their financial support (Cuervo-Cazurra et al., 2019).

Thus, studies that account for country development levels in their investigations of the relationships among globalization, development and corruption commonly find that relationships will hold only for developed countries, or alternatively, developing countries. Even when relying on simple binary categories (e.g., developed vs. developing, industrialized vs. emerging, or OECD vs. non-OECD countries), studies have repeatedly found different, even reversed, results for relationships across stages of development. Consequently, many studies qualify their conclusions within this development-based resource dependence contingency (e.g., Aron, 2000; Voyer & Beamish, 2004).

The stage of development is even more compelling, though, in that it is easily conducive to more than just two categories that respond better empirically (Allred et al., 2017; Bryant & Javalgi, 2016) and, is further reified in World Bank data. Indeed, Avnimelech, Zelekha, and Sharabi (2014) discovered that adopting the categories of developed vs. non-developed countries was insufficient for understanding the impact of corruption on entrepreneurship, which compelled them to exclude transition economies from their analysis. Asongu (2012) also recognized that binary categories limited the understanding of globalization relationships and provided evidence that development can be a key contingency when the four levels of low, lower-middle, upper-middle, and high income categories are employed. Our own findings based on these four development stages, which distinguish lower-middle from upper middle income countries, reveal important variance in globalization dynamics that cannot be captured when using the more encompassing “middle income” classification used, for instance, by Doner and Schneider (2016).

## INSTITUTIONAL CONTINGENCIES: THE DIMENSIONS OF GLOBALIZATION

Institutional theory, with its emphasis on macro-level change phenomena, is particularly well suited to shed light on globalization, whose inherent properties lie in the diffusion and adoption of global values, practices, and policies (Erez & Gati, 2004; Meyer et al., 1997). Importantly, institutional theory turns our attention to the notion that exogenous pressures for change and the search for legitimacy (not just technical efficiency) (Meyer & Rowan, 1977) determine organizational and social behaviors (DiMaggio & Powell, 1983). Change is further argued to result from isomorphic pressures towards conformity (DiMaggio & Powell, 1983; Meyer & Rowan, 1977).

The sociological institutional tradition (DiMaggio & Powell, 1983; Scott, 2001) varies in its terminology from the institutional economics tradition (North, 1990), but the two taken together provide an expanded lens for categorizing dimensions in globalization. Beginning with the sociological framework, institutions can be characterized as regulative, normative and cognitive (Scott, 2001), or responses to coercive, normative or mimetic pressures (DiMaggio & Powell, 1983). Regulative forces consist of rules, laws, sanctions and incentives, and they respond to expedience or coercive pressures. Normative forces encompass those that bestow honor or avoid shame, and can include certifications, standards, or accreditation; they draw on moral governance, and promote responses of social obligation or to normative pressures. Cognitive forces build on shared understandings and are reflected in common beliefs that support responses of conformity, or to mimetic pressures.

As the boundaries between the normative and cognitive dimensions of institutions are often blurry, Zimmerman and Zeitz (2002) argued for combining them under a single umbrella. This approach is further consistent with the institutional economics’ definition of institutions as either formal, i.e., reflected in rules that are readily available and generally written, or informal (North, 1990), i.e., “based on implicit understandings and therefore not accessible through written documents or necessarily sanctioned through formal positions” (Zenger, Lazzarini, & Poppo, 2001: 278). In their treatment, Pfarrer et al. (2008) combined insights from both the sociological and economic perspectives on institutions. They highlight the coercive character of regulative/formal institutions, defining them as driven by “involuntary compliance”, and alternatively, relate normative-cognitive/informal forces to “voluntary compliance”.

While institutional theory has become the primary lens to study IB phenomena, it has been incorporated largely in favor of the institutional economics perspective (Henisz & Swaminathan, 2008) which depicts institutions as given, or background conditions (Koning et al., 2018), and explains *why* organizations behave the way they do. In concert with this economics tradition, globalization in the IB literature has mostly been characterized as an economic phenomenon, consequently understating the many other ways interconnections can be formed (Lalountas et al., 2011; Meyer et al., 1997). But, because globalization is by nature a force that enacts processes of change, incorporating the less-static sociological institutionalism, which stresses a wider range of forces and responses, can serve as a better foundation for investigating both the *why* and *how* organizations and societies change in response to different forms of globalization (Koning et al., 2018). Recognizing a greater variety in globalization also answers recent calls to consider globalization as a multi-dimensional phenomenon (Akhter, 2004; Bryant & Javalgi, 2016).

Following this recommendation, Dreher (2006) composed the increasingly popular *Konjunkturforschungsstelle*, or KOF, index to measure different facets of globalization, specifically the three dimensions of economic, social, and political globalization. This measure was developed with Holm and Sorensen’s (1995) definition of globalization in mind, i.e., with the view that globalization should be conceptualized along three dimensions reflecting a nation’s economic, political, and socio-cultural interdependencies and interactions with other nations. These different dimensions correlate well with different institutional facets in countries, i.e., economic, social, and political (Meyer et al., 1997), and, as we discuss below, may contribute differently to the processes of development and corruption.

*Economic globalization* measures the “long distance flows of goods, capital and services as well as information and perceptions that accompany market exchanges” (KOF, 2013: 1). Contracts and the search for transaction cost minimization are central in economic globalization, and are usually represented in the formal, or regulative/coercive, dimension of institutions.

*Social globalization* is defined as “the spread of ideas, information, images and people” (KOF, 2013: 1), and thus tends to unfold informally (Cuervo-Cazurra et al., 2019) through the normative and cognitive pressures which underpin the diffusion and adoption of global values via social influence and peer effects (Cannizzaro, 2020; Galang, 2012). While rather neglected in IB research (Clegg, 2019), this aspect of globalization is gaining increasing interest. Kautto (2018: 397) noted that “social influences in international business transmitted through interorganizational and personal social networks create strategic value for the process of internationalization and increase the speed and performance of internationally operating firms”.

Lastly, *political globalization* is “characterized by a diffusion of government policies” (KOF, 2013: 1). Although institutional economics typically defines political forces as informal, political globalization regularly inhabits both formal and informal mechanisms. Government policies, for instance, can be transmitted through coercive pressures that underlie an *ex post* process of bargaining, such as international treaties (see Appendix). On the other hand, political forums can serve as normative-mimetic pressures of *ex ante* social influence (Cannizzaro, 2020: 830), such as peer effects that arise from membership and participation in supranational organizations (see Appendix). In other words, while economic globalization is driven by formal pressures and social globalization by informal pressures, political globalization encompasses both formal and informal mechanisms, such as respectively reflected in the KOF political globalization items “International treaties” and “Membership in international organizations”. This duality implies that political globalization may influence countries differently based on whether its driving mechanisms are formal or informal.

Accordingly, akin to institutional dimensions (Koning et al., 2018), globalization dimensions should also be conceptualized as interdependent. Social globalization, for instance, can influence political globalization, such as when global pressures for environmentalism (Meyer et al., 1997) produce national policy changes (Aïssaoui & Fabian, 2015). Economic globalization “can also be facilitated by informal institutions that enable the transfer of information, the building of trust and the curtailing of misbehavior” (Cuervo-Cazurra et al., 2019: 622), or by the “social networks embedded between businessmen and government officials that are utilized by firms to achieve preferential regulatory treatment” (Galang, 2012: 432).

In addition to these interdependent effects, the three dimensions of globalization also produce unique effects, hence the need to clearly distinguish each of these dimensions, i.e., to investigate the potential for each dimension to uniquely affect organizational and social phenomena. For instance, corruption is increasingly argued to be more effectively addressed through normative-cognitive pressures than through regulations (Allred et al., 2017; Pfarrer et al., 2008). This focus on norms reflects the evidence that informal institutions have more lasting effects than formal ones (Peng et al., 2017). We further note that governments regularly attempt to leverage institutions in unique ways to support their national and policy goals. For instance, China has regularly relied on a strategy aimed at fostering economic globalization, while simultaneously limiting factors of social globalization by blocking external influences for social change (Faure & Fang, 2008).

In summary, all three globalization dimensions are likely to relate to development and corruption through different mechanisms. Judge et al.’s (2011) study represents one of the few efforts to incorporate these dimensions to examine how they uniquely affect, or are affected by, corruption. The authors found that social and political globalization were significant antecedents to various corruption outcomes, and that economic globalization was both an antecedent and an effect of varied levels of corruption. Lalountas et al. (2011: 637) concluded after finding variance in their sample that, “Ignoring this differential impact of globalization may lead to incorrect inferences that guide to ineffective policies”.

Accordingly, in this study we investigate our central interest in globalization’s effects by first acknowledging the fundamental uncertainty over both the direction and valence of effects in the globalization–development–corruption nexus, as represented in Table 1. Second, we contend that employing stronger methods (longitudinal and causal) and greater granularity (contingencies) can help resolve some of these contradictions and provide a new starting point for theory elaboration, with an improved recognition of boundary conditions.

## RESEARCH DESIGN

**Figure 1:**
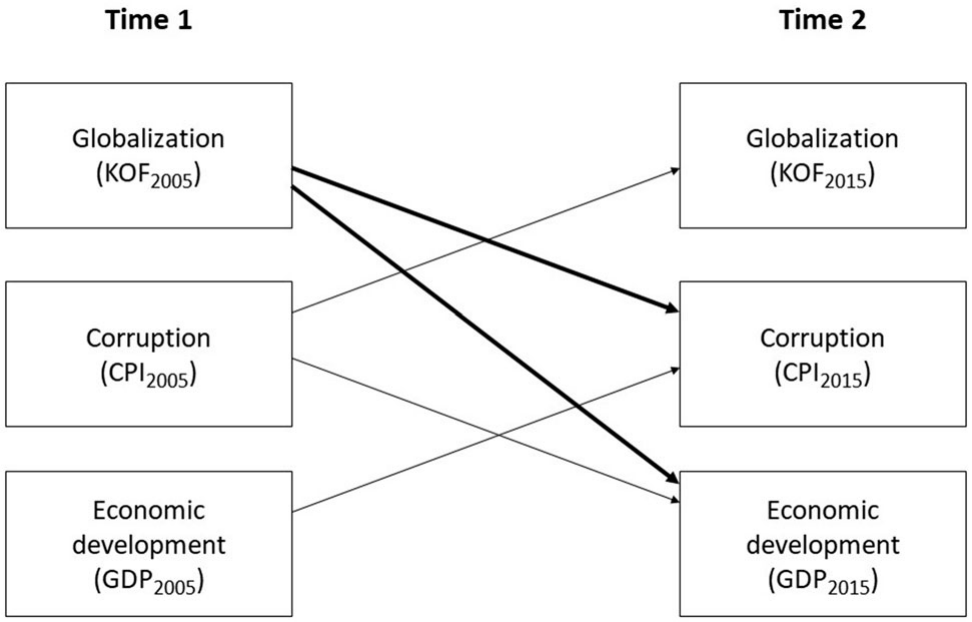
Main perspectives on the relationships among globalization, economic development, and corruption: Main model.

Based on the above theoretical perspectives, we develop a longitudinal, integrated model linking globalization, development, and corruption as illustrated in Fig. 1. This model then provides a basis for testing the contrasting perspectives subjecting them to the contingencies of country stages of development (low, lower-middle, upper-middle, and high income) and the institutional dimensions of globalization (economic, social, and political).

## VARIABLES AND MEASURES

*Globalization* was measured using *KOF* indices for each country, which offer the decided benefit of providing both a level of *overall globalization* (*KOF*), and a decomposition into the three dimensions of *economic* (*KOFE*), *social* (*KOFS*), and *political* (*KOFP*) globalization, with items detailed in the Appendix Table 9. The index arises from the work of Dreher (2006) and is regularly updated and made available by the KOF Swiss Economic Institute. Countries are ranked on these dimensions based on a scale from 0 to 100. Data are available on an annual basis for 207 countries for the period 1970–2015 (KOF, 2017). Potrafke (2015) provides an extensive review on the utility of the KOF index citing over one hundred studies employing the metric; consistent with our methods here he suggests controlling for reverse causality. More recently, Didzgalvyte-Bujauske, Pereira and Osteikaite (2019) relied on this index to find economic globalization by developing countries impacts their economic growth negatively in the short run, but positively in the long run.

*Economic development* was measured by the World Bank gross domestic product per capita (*GDP*), a macroeconomic indicator commonly used to assess the level of wealth in a country (World Bank, 2017). We deliberately chose the nominal GDP instead of real GDP, as the latter corrects for inflation. Given that inflation is highly correlated with corruption (Akça, Ata, & Karaca, 2002), the use of real GDP would deflate the effects of corruption. Furthermore, consistent with the common practice in social research of distinguishing countries based on their stage of development (Bryant & Javalgi, 2016), we used the World Bank’s categorization for each country as *low*, *lower*-*middle*, *upper*-*middle*, or *high income*.

*Corruption* was measured using the Corruption Perception Index (*CPI*), a composite index developed by Transparency International, using experts’ perceptions on the level of corruption in a country. Transparency International (2017b) continuously evaluates and updates the CPI. The index is argued to be the most comprehensive and robust measure of corruption (Voyer & Beamish, 2004), with convergent validity to other measures, such as the World Bank Control of Corruption (Judge et al., 2011).

### Sample

Our sample was constrained by the CPI data, as it covered the smallest number of countries among the three data sources. Furthermore, because the index and data collection process stabilized only after 2000, we restricted our study to the most recent data to preserve longitudinal comparability. Finally, as a period of 10 years is suitable to capture changes at the macro level (Aron, 2000), we collected data for the years 2005 and 2015 for each of the three variables under study.

Our study covers a total of 173 countries. However, depending on data availability, some models were tested with a smaller sample size (minimum *N* = 154). The stage of development was collected for each of these 173 countries for the year 2005 with the following distribution: (1) 52 low income countries, (2) 49 lower-middle income countries, (3) 34 upper-middle income countries, and (4) 38 high income countries (see Tables 2). Table 3 provides means, standard deviations, and correlations.

**Table 2:**
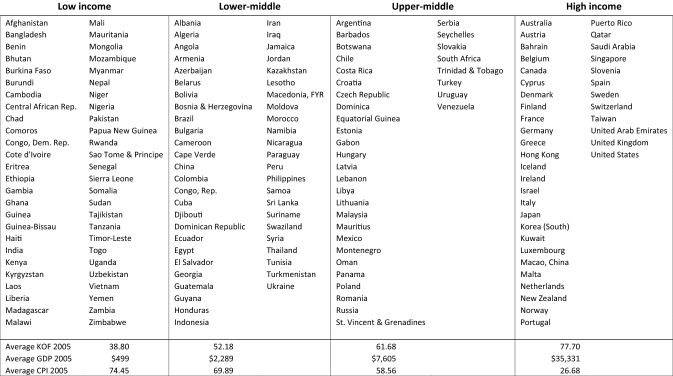
List of countries by stage of economic development in 2005

**Table 3:**
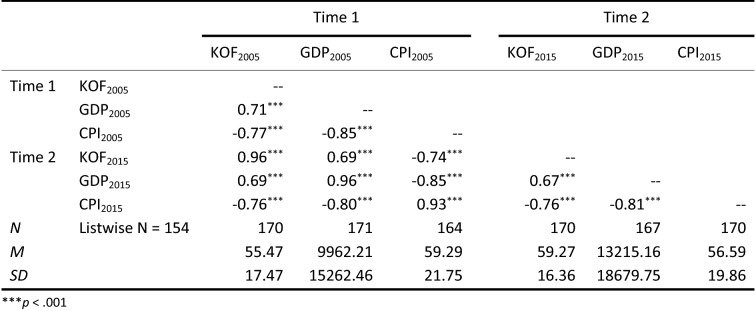
Mean, standard deviation, and cross-sectional and longitudinal correlations

### Analytical Approach

Calls for greater use of longitudinal methods reflect the importance of teasing out the dynamic “change in X leads to a change in Y” imperative of causal evidence (Akhter, 2004; Aron, 2000; Asongu, 2012). The cross-lagged panel design, also known as longitudinal structural equation modeling (SEM), tests a hypothesized pattern of directional and non-directional relationships among a set of observed and unobserved variables (MacCallum & Austin, 2000), and as such provides more evidence of causal precedence than does any type of cross-sectional design (Lang et al., 2011).

While a sample size of 200 is a common goal for SEM research (Bentler & Chou, 1987), a smaller sample can be used when three conditions are met: (1) the models have no latent variables, (2) all loadings in the models are fixed, usually to one, and (3) the models are based on strongly correlated variables (Kenny, 2020). All three conditions were met in our analyses.

Cross-lagged panel design analyses have their own limitations. Notably, longitudinal analyses should ideally examine three time periods to improve our appreciation of causal dynamics (Cole & Maxwell, 2003). However, as the number of time periods increases, so must the sample size to address the larger number of parameters to estimate. While our sample size was suitable for two time periods, expanding our analyses to three time periods would have resulted in strongly biased fit indices, and thus compromised the validity of our findings.

The cross-lagged panel method also provides the least biased estimates given the considerable need to account for the lagged, reciprocal causation that accompanies endogeneity (Allison, Williams, & Moral-Benito, 2017). This strength arises from testing and comparing competing models to explore plausible causal linkages among the variables (Cole & Maxwell, 2003). Cross-lagged methods also allow investigating simultaneity and mutual and reversed effects, all identified to be major methodological shortcomings in cross-country growth studies (Aron, 2000).

**Figure 2:**
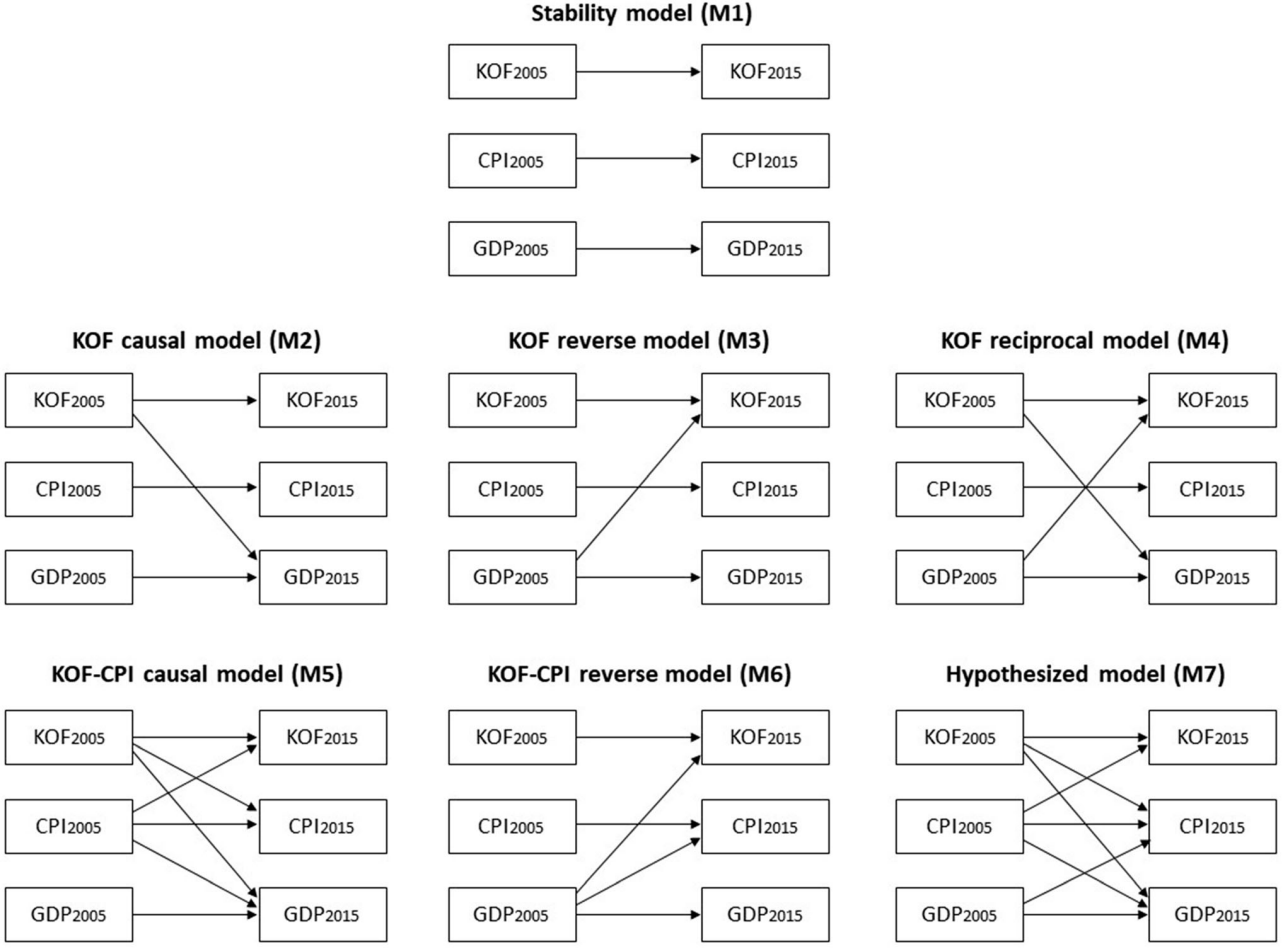
Relationships among globalization, economic development, and corruption: Seven nested models.

Under this approach, investigating the relationships among globalization, development, and corruption produces seven different nested models, as illustrated in Fig. 2. The first model, called the *stability* or baseline model (M1), assesses only the autoregressive effects over time of each variable, which assumes there are no causal relationships between one variable and another. Causality is examined with the next models (M2–M7), and we distinguish two different groups. The first group, Models 2 through 4, examines only the effects of globalization (*KOF*) on development: the *KOF causal model* (M2) assesses the cross-lagged effects from globalization to development; the *KOF reverse model* (M3) tests the reverse, less commonly developed, perspective whereby development spurs globalization; and the *KOF reciprocal model* (M4) combines paths from both M2 and M3.

The second group (Models 5 through 7) mirrors these tests but introduces cross-lagged paths from and to corruption (*CPI*) to test the effects of corruption over time as well as its response to changes in globalization and development levels. The *KOF*-*CPI causal model* (M5) estimates the cross-lagged paths from both globalization and corruption to development, along with the mutual effects between globalization and corruption; the *KOF*-*CPI reverse model* (M6) tests the reversed effects from development toward globalization and corruption. We did not test a *KOF*-*CPI reciprocal model*, which would combine M5 and M6, as this model would be fully saturated. Finally, the *hypothesized model* (M7) includes the paths identified by prevailing perspectives suggesting that (1) globalization affects both corruption and development, (2) corruption affects both globalization and development, and (3) development affects corruption.

Consistent with the cross-lagged methodological approach, we then assessed the relative strength of each model. The chi-square difference test assesses the change in fit upon the release of constraints to balance parsimony with fit (Kline, 1998). If a model with added paths is not found to be statistically different from a more parsimonious model, the latter is retained; conversely, if added paths create a statistically improved model, the more complex model is retained. SPSS Amos 23.0 assessed the model fits with a maximum likelihood estimation method. Following the recommended practice for longitudinal SEM, all cross-lagged models include measurement error covariances across time, based on the assumption that errors of repeated measures co-vary (Kline, 1998). All covariances were modeled, regardless of statistical significance, to account for common causes not included in the models.

## RESULTS

### Globalization, Economic Development, and Corruption: Best Model

Correlation results show that our three variables are significantly related, both within and across the two time periods under study (see Table 3). Furthermore, the three phenomena are rather stable over time (KOF 2005–KOF 2015 *r* = 0.96, GDP 2005–GPD 2015 *r* = 0.96, and CPI 2005–CPI 2015 *r* = 0.93).

The results from our cross-lagged analyses for these relationships at the global level are reported in Table 4. Following previous recommendations (Little et al., 2007), we first assessed the fitness of these models based on the chi-square test, the root mean square error of approximation (RMSEA), the Bentler–Bonett Index or Normed Fit Index (NFI), and the Comparative Fit Index (CFI) tests. Generally, cutoff values of 0.10 for RMSEA (MacCallum, Browne, & Sugawara, 1996), and 0.95 for CFI and NFI (Hu & Bentler, 1999) are used to evaluate the goodness of fit of a model.

**Table 4:** Globalization, economic development, and corruption at the global level: Models fit indices

	χ^2^	df	P	CMIN/df	NPAR	NFI	CFI	RMSEA
**M1- stability model**	16.76	6	0.100	2.79	21	0.989	0.993	0.10
**KOF models**								
M2 – KOF Causal	16.76	5	0.005	3.35	22	0.989	0.992	0.12
M3 – KOF Reverse	15.78	5	0.008	3.16	22	0.990	0.993	0.11
M4 – KOF Reciprocal	15.77	4	0.003	3.94	23	0.990	0.992	0.13
**KOF-CPI models**								
M5 – KOF-CPI Causal	1.44	2	0.487	0.72	25	0.999	1.000	0.00
M6 – KOF-CPI Reverse	15.51	4	0.004	3.88	23	0.990	0.993	0.13
**M7 – hypothesized model**	1.16	1	0.282	1.16	26	0.999	1.000	0.03

The results in Table 4 highlight four main trends. First, the stability model (M1) shows poor fit, indicating that even though globalization, development, and corruption are stable over time, changes in these variables need to accommodate other variables in the model. Second, Models 2 through 4 also have poor fits, with RMSEA values all above 0.10. When the corruption cross-lagged paths are introduced, two additional main findings emerge. The reverse models are poor at explaining the data, whether we exclude (M3) or include corruption (M6). However, introducing the effects of corruption substantially improves the models: M5 (RMSEA = 0.00), which introduces the cross-lagged effects of corruption, shows a significantly better fit than M2 (RMSEA = 0.12). The hypothesized model (M7) also shows a good fit, with a RMSEA of 0.03.

Next, using the chi-square difference test, we compared the models to identify the one that best fits the data. Table 5 provides results from this analysis. The cross-lagged paths in the KOF models do not improve variance explanation over the stability model autoregressive effects. When cross-lagged paths are added to include corruption, the stability model loses its fitness advantage as the M5 KOF-CPI causal model is significantly better at explaining the data than both the stability model and KOF models. Finally, the hypothesized model M7 is compared to the two best models, namely M1 and M5. The hypothesized model does not significantly improve upon the more parsimonious KOF-CPI causal model (M5), so M5 is retained.

**Table 5:** Globalization, economic development, and corruption at the global level: Model comparison

	CMIN Δ	df Δ	*P*
**Stability model vs. KOF models**			
M1 Stability vs. M2 KOF Causal	0.00	1	n.s.
M1 Stability vs. M3 KOF Reverse	0.98	1	n.s.
M1 Stability vs. M4 KOF Reciprocal	0.99	2	n.s.
**Stability model vs. KOF-CPI models**			
M1 Stability vs. M5 KOF-CPI Causal	15.32	4	0.005
M1 Stability vs. M6 KOF-CPI Reverse	1.25	2	n.s.
**KOF models vs. KOF-CPI models**			
M2 KOF Causal vs. M5 KOF-CPI Causal	15.32	3	0.005
M3 KOF Reverse vs. M6 KOF-CPI Reverse	0.27	1	n.s.
**Hypothesized model**			
M7 Hypothesized vs. M1 Stability	15.60	5	0.01
M7 Hypothesized vs. M5 KOF-CPI Causal	0.28	1	n.s.

**Figure 3:**
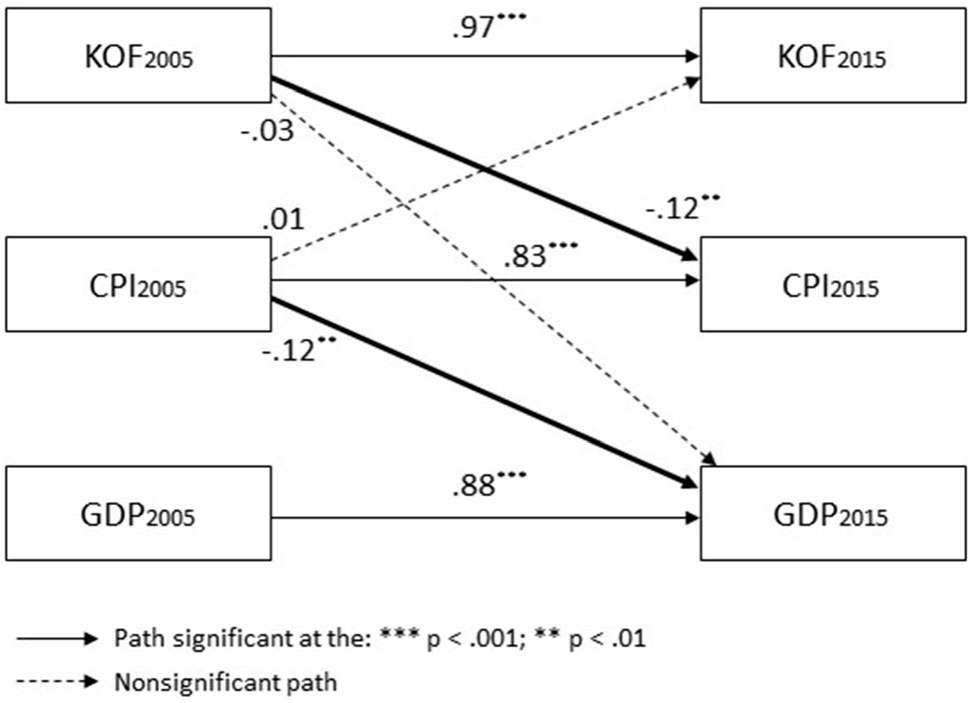
Relationships among globalization, economic development, and corruption: Best model.

In sum, our analyses indicate the KOF-CPI causal model, while close to our hypothesized model, best fits the data. In addition, these analyses help identify which effects are (non)significant in any of these models. The significant parameter estimates of the best model (see Fig. 3 and Table 6) suggest a sequential dynamic whereby globalization decreases corruption (KOF_2005_ → CPI_2015_: *β *= −0.12, *p *= 0.006), which then positively impacts development (CPI_2005_ → GDP_2015_: *β *= −0.12, *p *= 0.004).

**Table 6:**
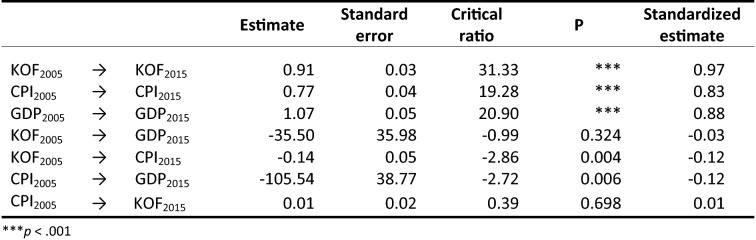
Globalization, economic development, and corruption at the global level: Best model parameter estimates

## ASSESSING THE ROLE OF THE COUNTRY’S STAGE OF DEVELOPMENT AND GLOBALIZATION DIMENSIONS

**Figure 4:**
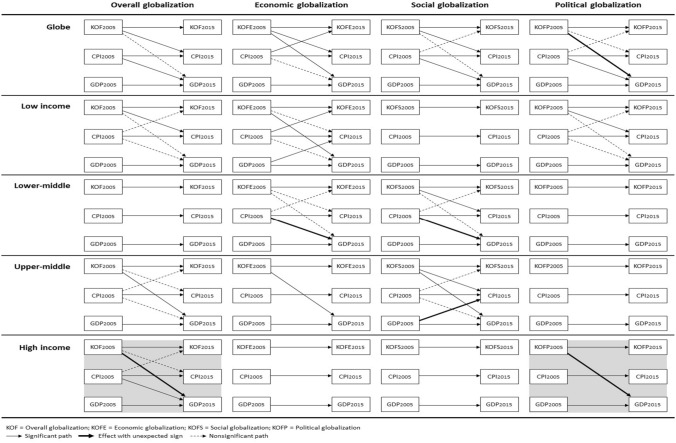
Best models by country stage of development and globalization dimension.

We first proposed that the relationships among globalization, development, and corruption, will vary based on the country’s stage of development – low, lower-middle, upper-middle, and high income. Further descriptive statistics for each of these categories are available upon request. Our second proposition contended that globalization can be decomposed into different dimensions that have dissimilar theoretical mechanisms for their effects. We repeated the analyses described in the previous section to accommodate these two conditions. Investigating these conditions resulted in 20 different iterations of this analytical process (five for the four stages of development and the global sample, multiplied by four for the three globalization dimensions and the overall globalization scores). Table 7 provides a summary of our results indicating the best model for each combination, along with the significant paths and the size of the autoregressive paths for our three variables of interest. Figure 4 is a graphical representation of these models with significant paths in solid lines. Those paths whose effect has a different sign than expected are highlighted in both Table 7 and Fig. 4.

**Table 7:**
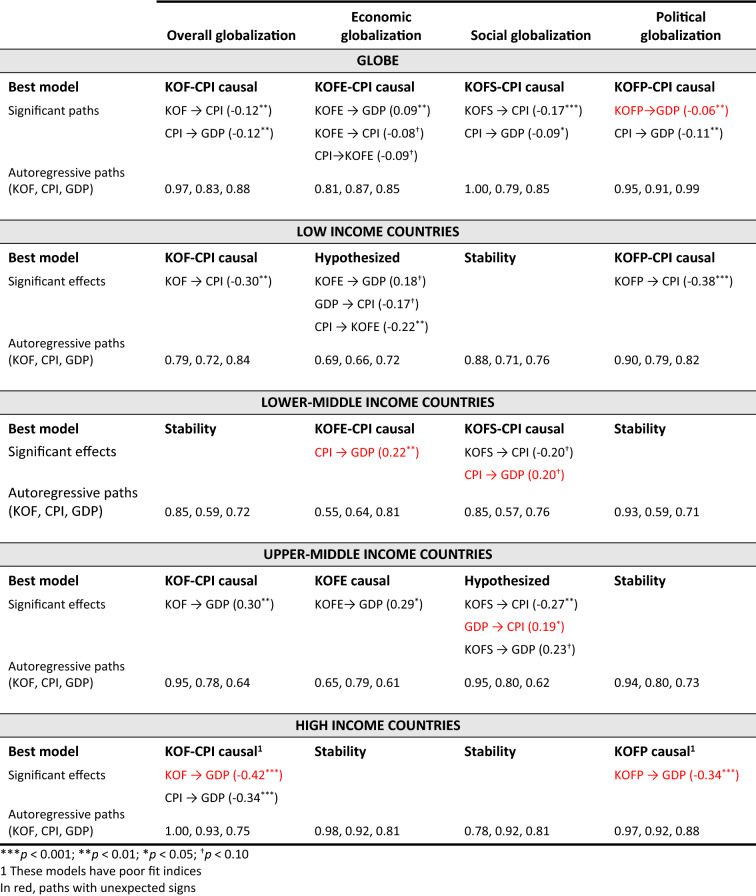
Summary findings best models by country stage of development and globalization dimension

Our findings support our propositions that the effects are contingent upon both the country stage of development, and the globalization dimension. Indeed, there is strong variance both in terms of which model best fits the data, and which paths are significant, which suggests that varied mechanisms are at play based on these two main conditions. Below, we detail our findings by country stage of development, highlighting their unique globalization trajectories.

### Sequential Dynamics at the Global Level

Our findings from the global sample suggest that globalization is key to understanding economic growth whether its impact is direct or indirect (affecting development through its interactions with corruption). The overall and social globalization models share similar sequential dynamics whereby globalization reduces corruption which, in turn, benefits development. Economic globalization, on the other hand, affects both development (*β *= 0.09) and corruption (*β *= –0.08) simultaneously. Finally, while political globalization affects development, the sign of this effect is contrary to expectations (*β *= – 0.06), suggesting countries do not benefit economically from increased political integration. This finding strongly recommends the necessity of investigating our boundary conditions, as further analysis reveals this relationship is primarily driven by the strong negative effect that political globalization has on development in high income countries (*β* = – 0.34). In fact, all of the relationships that are significant at the global level generally do not hold across the four different stages of economic development.

### Low Income Countries and the Importance of Coercive Forces

Low-income countries seem to be the most undermined by corruption, as it both prevents globalization from positively effecting development, and hinders globalization itself, providing evidence of *sand in the wheels* of globalization under the economic globalization model (CPI → KOFE *β *= – 0.22). However, globalization does impact development positively, first indirectly by having the virtuous effect of fostering the adoption of corruption-reducing practices (*diffusion* mechanism: KOF → CPI *β *= – 0.30 and KOFP → CPI *β *= – 0.38 under the overall and political globalization models respectively); second by providing, under the economic globalization model, new, legitimate, source of revenues which can substitute for the gains from corruption (*compensating* mechanism: GDP → CPI *β *= – 0.17).

Furthermore, this group of countries responds positively to coercive pressures, which supports arguments that formal forces, such as international treaties (Olabisi, 2019), may be most effective at jumpstarting countries plagued with institutional voids: both economic and political globalization significantly impact this group, whereas the social globalization model exhibits no significant effects. This finding is consistent with Brandl et al.’s (2018) claim that developing countries with institutional voids are more likely to focus their efforts on formal institutions. It further suggests that what may be at play in these political dynamics reflects the formal aspect of political influence more so than its informal facet. We tested this possibility with a separate analysis that revealed that, for this group, it is the formal aspect of political globalization (i.e., where sanctions are attached for noncompliance) that was affected the most in low-income countries: the scores for the item “International treaties,” which reflect the formal aspect of political globalization, grew on average by 8.24 points between 2005 and 2015, compared to 6.28 for its informal aspects as reflected in membership to various political organizations (see Appendix in Table 9).

Zenger et al. (2001) provide various insights on the unique versus interactive working of formal and informal institutions. First, the existence of inertia causes slow change, thereby creating a lag between the implementation of formal institutions and effective changes in informal institutions. This further suggests that formal institutions may set the stage for changes in informal institutions. Third, they argue it may be easier to disrupt existing formal institutions. Our findings from both low-income countries and more developed countries provide strong support for these claims. However, these statements reflect a widely shared “implicit conceptualization of informal institutions as substitutes for formal ones, i.e., informal institutions are strong when formal pro-market institutions are weak” (Cuervo-Cazurra et al., 2019: 622), which is evidenced in the claim that, “formal institutions are not only unnecessary but also damaging to the formation and operation of informal elements” (Zenger et al., 2001: 285). While there is value in this “substitution” perspective, based on our evidence from low-income countries who respond to formal pressures but not to informal ones, our findings from the more developed group of lower-middle and upper-middle-income countries favor, instead, the “complementarity” perspective (Cuervo-Cazurra et al., 2019) whereby institutional arrangements work in more sophisticated ways (Doner & Schneider, 2016: 610).

### Lower-middle Income Countries and the Rising Effectiveness of Normative-cognitive Forces

Lower-middle-income countries offer our only evidence that conditions may arise for *grease the wheels* activities, where corrupt practices may be used to overcome institutional voids (CPI → GDP *β *= 0.22 and 0.20 under the economic and social globalization models respectively). Furthermore, normative-cognitive forces are beginning to show their effectiveness as the social globalization model is now significant, though only in relation to corruption. Indeed, despite corruption’s seeming positive contribution to development, our findings suggest corruption is simultaneously reduced from social globalization (KOFS → CPI *β *= – 0.20). This may indicate a transitional stage as domestic actors begin to lobby and build the necessary institutions to combat corruption rising from their awareness from social globalization (Tihanyi & Hegarty, 2007).

### Upper-middle Income Countries and the Complementarity of Formal and Informal Institutions

Upper-middle-income countries begin to witness the results of the policies and strategies aimed at attracting new businesses and investments, as evidenced by the increased wealth from most globalization dimensions (KOF → GDP *β *= 0.30; KOFE → GDP *β *= 0.29; KOFS → GDP *β *= 0.23). At the same time, this economic performance may have been achieved partly through previous investments in corruption (lower country stage’s grease the wheels), which would further explain the need to obtain a return on such investments by engaging in rent-seeking operations (GDP → CPI *β *= 0.19 under the social globalization model).

Further, as these countries are attracting more foreign investors, they are also increasingly exposed to corruption-reducing norms and values, as evidenced not only by diffusion mechanisms from social globalization, but also by the increasing strength of these normative-cognitive pressures (KOFS → CPI *β *= – 0.27 compared to – 0.20 for lower-middle-income countries). This finding supports Aguilera et al.’s (2008) view that, in the more mature phases of economic cycles, demand for accountability increases.

### High Income Countries and the Undermining Effects of Normative Pressures

Findings from high-income countries suggest that corruption is increasingly less relevant as countries become relatively affluent. Indeed, we found no role for the corruption variable in any of the economic, social, and political globalization models. It is only with the cumulative effects from all the globalization dimensions in these countries that evidence arises that corruption undermines development (CPI → GDP *β *= – 0.34 under the overall globalization model).

Political globalization becomes conspicuously central at this level of development, consistent with Lalountas et al.’s (2011) and Hoekman and Nelson’s (2018) findings that high-income countries emphasize the political dimension of globalization. However, political globalization undermines development (KOFP → GDP *β *= – 0.34), which, though surprising, elicits multiple explanatory possibilities. First, “Capital mobility gives employers a credible threat: accept lower wages, or else we move abroad” (Rodrik, 2018: 21). Further, greater political globalization often results in globally harmonizing regulations which tend to favor multinational corporations, and “industries/interest groups that are welfare-reducing” (Hoekman & Nelson, 2018: 40). A third explanation is provided by Levie and Autio (2011) who found that higher regulatory burdens when paired with the effective rule of law – something associated with high stages of development – was associated with lower rates and prevalence of strategic entrepreneurial entry, a central mechanism associated with economic growth.

Lastly, the neo-institutionalist argument that institutional change is motivated not just by technical or efficiency criteria but also by a search for legitimacy (Meyer & Rowan, 1977) may further explain this outcome. It may just be that the search for increased legitimacy gained from new political connections may expose high-income countries to pressures to implement global policies, standards, and practices which affect the existing economic equilibrium and therefore undermine the economy. Thus, it may just be that at this level of development, political globalization, when driven by a need for legitimacy in the global arena, may be associated with an economic cost. Further analyses show that, indeed, contrary to low-income countries who benefit from the efficiency-enhancing mechanisms from increased *formal* political globalization, those high-income countries who relied primarily on the legitimacy-enhancing mechanisms from *informal* political globalization – with an increase by 6.76 points on average in the membership items compared to 2.58 in the formal aspects – did witness a decline in their economic performance – on average – 9% in GDP. We return to this issue in the discussion below.

## DISCUSSION, IMPLICATIONS, AND FUTURE RESEARCH

The central role of globalization in driving development continues to fill scientific journals, as policymakers and business decision-makers alike are keenly interested in understanding the state of a country’s economy and its future. Further, as corruption is strongly featured as an intervening factor in this relationship, we opened this research with an integrative review of perspectives that animate four unsettled disputes in the globalization–development–corruption nexus, noting that these variables appear to sometimes cause, and sometimes be caused, by each other; sometimes for good, and sometimes for bad.

It is these “sometimes” that we argued need greater elaboration in order to better guide policy and business decision-making. Specifically, we proposed that these “sometimes” may in fact hide the existence of consistent patterns of differential globalization impacts. With the aim of identifying these differential impacts, we pursued grouping dynamics in countries by their stage of development (low, lower-middle, upper-middle, and high-income), and opening the globalization construct across its three different dimensions (economic, social and political) to discern how these resource dependence and institutional contingencies may drive different globalization trajectories. In addition, we incorporated recent advances in statistics with cross-lagged panel methods both to improve causal insights and to account for endogeneity and simultaneity issues (Aron, 2000; Asongu, 2012; Dreher, 2006).

In so doing, we address calls to shed light on the determinants of institutional change as policy and business decision-makers depend upon it to “predict, plan for and adapt to such changes” (Koning et al., 2018: 251). We further respond to the need to unpack the different dimensions of globalization as the insistence on its economic facet is detrimental to both efficient policy making (Allred et al., 2017; Pfarrer et al., 2008) and to our understanding of rising anti-globalization sentiments (Hoekman & Nelson, 2018; Ozawa, 2019). The use of a cross-lagged panel design further addresses continued calls for assessing institutional change with a longitudinal approach (Brandl et al., 2019; Dimant & Tosato, 2018).

Our findings indeed identify startling differences in impacts from the economic, social, and political dimensions of globalization, notably in relation to the country’s stage of development. These findings thus support our main contention that without an accommodation for both these contingencies, the pooling of results on a global basis could be highly misleading. Importantly, the integrative approach taken in this study – whose decided advantage is the comparability of the results owed to the use of the same data, the same time period, and the same method – provides a platform for future research, and allows us to make two substantive contributions which further provide new directions for policy-making.

## BOUNDARY CONDITIONS RELATED TO EXISTING PERSPECTIVES

Our literature review pointed to four main globalization debates (see Table 1), each proposing different theoretical relationships among globalization, development and corruption. We took a contingency approach, arguing that some generalizability may be found in these differential impacts by identifying two conditions – i.e., resource dependence and institutional mechanisms – that bound these influences. This integrative approach is not only consistent with the main tenet of contingency theory that “no one size fits all” (Donaldson, 2001); it also allowed us to respond to each of these debates without the assumption that the same model must equally explain relationships across contexts. Table 8 summarizes our findings.

**Table 8:**
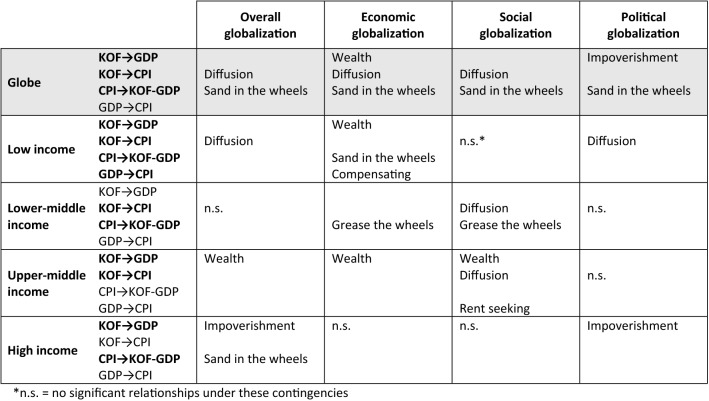
Theoretical perspectives by country stage of development and globalization dimension

It is worth noting that even though the two contingencies and three variables evaluated in this study may not comprehensively account for the complexity of interdependence between globalization dynamics and other institutional (e.g., governance, democracy) or resource dependence factors (e.g., technology frontier, human capital), we believe that this approach is a useful conceptual tool to analyze why globalization does not always produce its desired effects.

### Wealth vs. Impoverishment

Our findings provide marginal evidence of impoverishment under increased globalization, and only in the one condition of increased political globalization by high-income countries. Despite arguments that globalization impoverishes countries through exploitation (Carr & Chen, 2001) or outsourcing (Krugman, 2008), we found no evidence supporting these perspectives. Specifically, because only increasing political globalization is related to impoverishment in high-income countries, the perspectives that either impoverishment through exploitation is occurring, or that greater outsourcing from economic globalization undermines development appear suspect. Accordingly, we turn to Granovetter’s (1985) notion of “embeddedness” to offer a different perspective on this unique trajectory.

Granovetter (1992: 9–10) elaborated on the complexity of societies, which incorporate multiple “equilibrium points” that “lock in” societies into particular structural arrangements. Changes in a particular institutional dimension are likely to generate a disequilibrium, which suggests that movement on one dimension can negatively affect other features of embedded relationships. Given that our sample countries experiencing greater impoverishment already reside in a high-income stage of development, then efforts towards institutional change (via tighter political integration) can only mean altering existing embedded ties that have already proven successful to their context.

We briefly review a set of high-income countries in our sample that hail from various regions in the world regarding this dynamic: Greece, Iceland, the United Arab Emirates (UAE), and Japan. Rodrik (2018) had similarly identified Greece with respect to the growth-dampening effects a country can experience from greater political globalization. With regards to Greece, Westaway (2012) provides a compelling historical account of the country’s economic challenges following its integration into the European Union (EU), noting that pressures for economic austerity policies – themselves strongly motivated by EU competitive considerations* vis-à-vis* globalization – were antithetical to the country’s culture of patronage and populism. Iceland experienced a similar trajectory: its application to the EU in 2009 – later withdrawn in 2013 – required Iceland to repay its debts to the UK and the Netherlands, and to implement structural changes to align Iceland’s economy with, among others, the EU’s burdensome capital movement restrictions or the imposition of quotas in the fishing industry, one of the main sources of Iceland’s wealth (Jones & Clark, 2013).

The UAE’s declining economic performance also coincides with an attempt from the country’s leadership to gain legitimacy from its global partners. In 2007, the UAE announced its national development plan to make “the country a world leader – that is to say, an economic power with political influence” (Country Watch, 2016). This plan entailed a series of political and institutional reforms focused on transparency and global and regional integration (Fanack.com, 2020). The common thread among these three country exemplars is best encapsulated in Japan’s “syncretism,” a term used to refer to the country’s disruption of existing normative and socio-cultural institutions from a restructuring of its formal institutions (Kushida, Shimizu, & Oi, 2013). Indeed, Japan’s declining performance, akin to Greece, Iceland, and the UAE, followed efforts at gaining global legitimacy through increased political globalization, which clashed with the “old” ways of doing things.

In sum, greater political integration exposes countries to pressures from international institutions towards globalizing their policies and practices. Sometimes, though, such policies clash with existing cultural orientations (Stiglitz, 2003), and their implementation may create a disequilibrium that will be reflected in economic decline, at least until the country finds a new “equilibrium point” (Granovetter, 1992). The adoption of integration policies in advanced economies should therefore be cognizant of this dynamic and consider the disruption of a functioning equilibrium that arises from harmonization. Similarly, governments must recognize that the legitimacy that can be gained from joining new political communities may have to be achieved to some degree at the expense of their development.

### The Ills of Corruption

Three debates relate corruption to globalization and development, namely, the *diffusion vs. contagion*, *compensating vs. rent*-*seeking*, and *sand vs. grease the wheels* theories. Here, we found no evidence that globalization, of any kind, was related to increased corruption in later periods. Further, although greater globalization does not reliably generate wealth, we do find strong evidence of a virtuous effect on corruption from institutional diffusion.

Lack of support of the spread of corrupt practices from globalization implies that the current perspective of institutional contagion may be incomplete due to a confounding effect/simultaneity problem, which in effect attributes to globalization what is instead based on a rise in rent-seeking opportunities from economic growth. As illustration, consider the results in Tables 7 and  8 on the significant dynamics in upper-middle income countries. Greater social integration decreases corruption. But, it also increases wealth. At this stage of development, greater wealth opens up profitable opportunities for rent-seeking, which incorporates greater corruption. This set of paths, which vindicates globalization as not the direct cause of corruption, was only able to be empirically uncovered by employing a rigorous causality test that could identify both simultaneity and confounding effects.

Importantly, we find that it is primarily through social globalization that the diffusion of corruption-reducing practices unfolds; political globalization reduces corruption only in low-income countries, and it is only at the aggregate level that economic globalization positively impacts corruption. This finding thus points to the need to rely more systematically on socialization policies in the fight against corruption, consistent with the view that coercion through regulations is often ineffective (Sharman, 2017). Alternatively, business transactions may help by contractually enforcing anti-corruption provisions allowing one party to breach the contract when evidence of corruption by the other party arises. Pfarrer et al. (2008: 387) noted that, “This type of industry self-regulation indeed may be more effective than traditional, formal deterrence methods”.

Lastly, we note the overwhelming support for the argument that corruption undermines development (evidenced in four models) and globalization (evidenced in two models). The *grease the wheels* theory holds only under two contexts, and only for lower-middle-income countries. Thus, clearly corruption is an impediment to development and global integration. As governments engage in economic integration, they should consider that absent corruption-reducing policies, their efforts to attract foreign investors will prove unsuccessful. However, caution is advised against any conclusion supporting the grease vs. sand mechanisms given the often unaccounted for, but extensive, illicit financial flows (Kar & Spanjers, 2015).

## DIFFERENTIAL IMPACTS OF FORMAL AND INFORMAL INSTITUTIONS ON ECONOMIC DEVELOPMENT AND CORRUPTION

Our second contribution consists of highlighting the distinct roles played by formal and informal institutions in development and corruption. Contemporary economic theory recognizes institutions as fundamental sources of development (Kim et al., 2010), as they represent limitations on economic, political, and social interactions, and can be either formal or informal (North, 1990).

### Distinguishing the Formal and Informal Mechanisms of Political Globalization

First, we premised our arguments around the views that economic globalization overwhelmingly tends to rely on formal mechanisms, while social globalization occurs through informal mechanisms, that is, implicit understandings such as norms and routines. However, while political processes are often depicted as an informal phenomenon (North, 1990; Zenger et al., 2001), our analysis of the data advocates for differentiating political globalization’s impacts by both its formal and informal mechanisms. The institutional duality of political globalization is evident in the items that compose this index (see Appendix in Table 9): the role of embassies, membership in international organizations, and participation in U.N. Security Council missions have a voluntary, informal, character (Pfarrer et al., 2008), whereas international treaties encompass formal, written provisions that are coercively enforceable.

By lumping all political globalization as representing informal institutions, the role that formal, enforceable, political forces can play in influencing some countries’ economic and corruption policies is lost; and the ability to formulate policies that acknowledge such differential impacts is similarly missing. By conceding that political globalization can be both formal and informal, we capture that political globalization may actually serve different functions, namely promoting efficiency versus legitimacy. In particular, low-income countries are most focused on enhancing their economic efficiency in global trade, and they tend to implement the *formal* aspects of political globalization in priority, which, in turn, positively impacts their economic development. Alternatively, informal mechanisms in political globalization than can increase global legitimacy are more notably pursued by high-income countries, which we find may be achieved at the cost of disrupting the existing economic equilibrium.

We therefore urge globalization scholars to consider not just the differential impacts from different globalization dimensions (Dreher, 2006; Judge et al., 2011), but also the potential for these dimensions to be driven by different institutional mechanisms, namely formal or informal. Doing so may further help in recognizing the role of institutional resilience and the necessary processes for designing different baseline rules to generate growth depending on the strength of a country’s formal institutions (Aligica & Tarko, 2014), as well as the risks from disrupting the existing economic equilibrium when priority is placed on the search for global legitimacy.

### Formal and Informal Institutions across Development Stages

**Figure 5:**
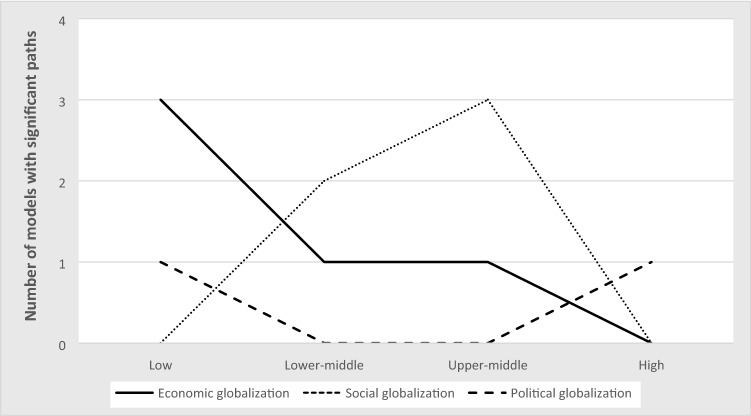
Countries’ responses to globalization forces.

Our study disentangles further the role of formal and informal institutional forces, and thus responds to calls to identify not only their unique effects on development and corruption, but also the conditions under which they are effective (Allred et al., 2017; Clegg, 2019; Cannizzaro, 2020; Koning et al., 2018). Figure 5 provides a visual depiction of these effects along the four stages of development.

Briefly, formal forces from economic globalization are effective at lower levels of income and lose their effectiveness with increased wealth. This trend suggests a negative relationship between wealth and the effectiveness of formal institutions, a relationship that does not hold for informal forces. Indeed, informal pressures, reflected in social globalization, are ineffective at lower levels of income and become increasingly effective with increased wealth, but only up to a certain threshold. Finally, political globalization displays a U-shaped trajectory that reflects the need for increased efficiency from low-income countries, notably to address their institutional voids (Olabisi, 2019), and the need for increased legitimacy from high-income countries. Thus, while there is increasing agreement that coercive pressures, here in the form of multilateral treaties and national policies (Di Pietra & Melis, 2016) are ineffective, notably to combat corruption (Ang, 2020; Sharman, 2017), our study provides a more nuanced appreciation, consistent with Allred et al. (2017) who recommend not discounting the possibility that certain contexts may respond favorably to formal compliance pressures.

These findings are central to shedding light on why globalization does not always result in improved wealth or reduced corruption, and more importantly why the notion of “universal best practices” (Aguilera et al., 2008: 481) is not just untenable; it also bears the risk of supporting ineffective policies (Lalountas et al., 2011) when the resource dependence and institutional contingencies suggest the conditions are not met for these policies to be successful. Clegg (2019: 112) laments that despite its potential to guide policymaking, institutional theory has been “relatively poor at making predictions in an international context”. We do agree with Clegg but also demonstrate how a more systematic consideration of informal forces can improve institutional theory’s predictive validity.

### Expanded Policy Recommendations

We address the above concerns with policy recommendations that draw from the sociological institutionalist tradition of reserving a central role for institutional actors, concentrating on those that drive regulative, normative and cognitive change (Lawrence, Suddaby, & Leca, 2009). International business research has recently drawn our attention to the role of actors in institutional change, recognizing that influential actors vary depending on the institutional context and stage of development (Brandl et al., 2019). Darendeli and Hill (2016), for instance, note that non-governmental and public organizations have little influence in the face of institutional voids. Doner and Schneider (2016) further attribute the slowness of institutional change in middle income countries to the fragmentation of their actors, which prevents the building of coalitions required to implement large institutional investments.

Despite these insights, it is still unclear “who should do the policing and exactly what in national policies should be policed” (Buckley, 2018: 198). Our results suggest some effective policies and identify actors able to drive positive institutional change that can raise development and lower corruption. In regard to employing formal economic and political globalization initiatives, the focus should continue on low-income countries. Actors at the supranational level (e.g., IMF, WTO) can be effective in these environments, especially in their ability to incentivize corruption-reducing behaviors via the provision of valuable resources. Soon after establishment, though, these actors’ ability to further change conditions is limited or even negligible (Das & DiRienzo, 2009; Koning et al., 2018), given coercive pressures often offer poor incentives for compliance in more developed countries (Nebus, 2019).

Our findings indicate that as wealth increases, then coercive pressures from formal institutions lose their effectiveness in favor of a stronger role for normative and cognitive forces. Accordingly, policies should shift from relying on those actors able to set and enforce rules and regulations toward those actors whose legitimacy provides them with the tools to influence norms and guide behaviors (see also Cuervo-Cazurra et al., 2019). Here, policymakers should thus both focus on addressing wealth-dampening behaviors by citizens and corporate elites, and augment the levers of social influence from actors such as MNEs and media. For instance, transparency initiatives can emphasize building social norms that combat the demand side of petty corruption, such as limiting cash payments (Ang, 2020), and publicly promoting and demonstrating both the individually and socially harmful effects from corrupt practices. Follow-on efforts could focus on the supply side of corruption and target corporate elites through peer influence and leadership (Pfarrer et al., 2008). Furthermore, MNEs’ central role in institutional change should be recognized, notably as they can influence formal institutional change via their direct impacts on the economy (Kwok & Tadesse, 2006), and their ability to pressure the state for regulatory reforms consistent with good governance (Akhter, 2004). Alternatively, these firms can also influence informal institutional change (Erez & Gati, 2004) by encouraging ethical behaviors (Nebus, 2019; Petricevic & Teece, 2019), specifically in those upper-middle-income countries where the impact of social forces is strongest.

Consequently, our findings call for policies that promote “socially minded MNE exemplars” (Nebus, 2019: 269) to combat the exploitation of corporate power and the more grand corruption that can expropriate country wealth (Abotsi, 2018). If MNEs’ influence appears to foster wrongdoing and unethical behaviors (Kaufmann, 2005), policies can alter incentives to reward social ends in firm strategies (Buckley, 2018; Medina, Bucheli, & Kim, 2019). Collaborations between MNEs and governments (Doner & Schneider, 2016) and between MNEs and supranationals (Ozawa, 2019) aimed at instilling anti-corruption norms may serve as vital channels of influence. Importantly, this MNE influence should be counterbalanced by the proper working of democratic institutions (Medina et al., 2019), such as those that support freedom by the media to expose corporate wrongdoing, or the ability for informed citizens to boycott (Wang & Li, 2019).

Finally, we noted that the highest income countries may be trading off some of their wealth-enhancing mechanisms in favor of legitimacy-enhancing initiatives when further pursuing political globalization. We related this finding to a problem of disequilibrium (Granovetter, 1985). Consequently, a gradual, medium-term, approach for political integration by these countries may be preferable to hastened efforts to join new political communities. Specifically, given joining new global political commitments may satisfy a search for greater legitimacy, these countries need to counterweigh these benefits to reduced economic efficiency. Here, more piecemeal transitions should offer opportunities to monitor and respond to these unexpected effects on the economy.

## LIMITATIONS AND FUTURE RESEARCH

### Methodological Limitations

While a cross-lagged panel design allowed us to provide a stronger test for causality among the relationships in the globalization–development–corruption nexus, the conservative results from a small sample size may overlook significant paths (Westland, 2010). Importantly, given the sample size requirements of structural equation models, we could not control for factors that have been commonly identified as influencing some of these relationships. For instance, Lalountas et al.’s (2011) assessment of the relationships among globalization, corruption and development controlled for both geographic proximity between countries, and the country’s human development index, and found these variables significantly related to their core relationships. Here, we have also substantially expanded our understanding of these relationships with our contingency approach, controlling for the country’s stage of economic development and the globalization dimension, as illustrated by our twenty models. Given more evidence from other studies (e.g., Judge et al., 2011), future research should strive to evaluate the related roles of variables such as geographic proximity, or other determinant control variables, to our findings here.

### Decoupling

We briefly suggested that the opacity of illegal financial flows obscures our understanding of the real dynamics linking globalization to corruption. Further, the tendency for advanced economies to be linked to such practices (Cuervo-Cazurra, 2018) is strongly at odds with their ranking on corruption indices. This suggests a tendency for decoupling, or the creation of gaps between formal policies and actual practices (Meyer & Rowan, 1977), which is even more important in that the actors most likely to engage in decoupling, i.e., MNEs (Petricevic & Teece, 2019) are also those who can most likely drive institutional diffusion. Future research investigating decoupling by MNEs in the context of the implementation of corruption-reducing and wealth-enhancing practices may shed light on some of these phenomena, notably as globalization has also developed new opportunities for such decoupled behaviors in the form of illegal financial flows.

### Country Categorization

We note that our categorization of countries based on their stage of development remains a rough proxy. Consider for instance that high-income countries are highly heterogeneous in terms of their economic models, encompassing, for instance, a subset of oil-exporting countries characterized by high levels of corruption. Such heterogeneity should be accounted for when interpreting our general lack of cross-lagged effects in both the economic and social globalization models for these countries. Future research should pointedly theorize and test for how different classifications (e.g., colonial ties, age of democratic institutions) may be as revelatory as this first cut in evaluating phenomena such as globalization dynamics. Allred et al. (2017) also recommend grouping tax haven countries under a separate category.

### Time

Another powerful development of this line of research could be a focus on the role of time on the observed impacts. Different periods based on short, medium, and long-term assessments may reveal some tempering or exacerbating effects. Aidt et al.’s (2008) study offers one such example in which models on corruption were competitively tested based on both short- and long-term growth rates. Increasing our analyses across different periods should ward off premature conclusions: “how long” it takes for a change in globalization, development, or corruption to translate into a change in other variables is an open question.

Moreover, even the proper period for change is likely itself contingent on different institutional contexts (Peng et al., 2017). As such. globalization theory would strongly benefit from questions such as “How long does it take for globalization to translate into more development or less corruption?” or “How long does it take for improved development to affect corruption and globalization?” Given our results, these questions will be sensitive to their contexts.

## CONCLUDING REMARKS

In conclusion, our findings provide the salutary evidence that the mixed and contradictory findings of prior research are likely indicative of very valid cuts of a more complex phenomenon, one that is highly contingent on context and the globalization process in action. Through the addition of just two contingencies – the country stage of development and the globalization dimension, we found compelling evidence that many of the contrary perspectives in our literature review indeed do appear to occur differentially across contexts. Researchers may take comfort in this assurance, but they should also take heed that the move from contentious debate to contingency-thinking requires increased effort in a priori theorizing practices.

## Appendix

See Table 9.

**Table 9:** Variables and weights of the KOF globalization index

Indices and variables	Weights (%)
**Economic globalization**	36
**(i) Actual flows**	50
Trade (percent of GDP)	21
Foreign direct investment, stocks (percent of GDP)	28
Portfolio investment (percent of GDP)	24
Income payments to foreign nationals (percent of GDP)	27
**(ii) Restrictions**	50
Hidden import barriers	22
Mean tariff rate	28
Taxes on international trade (percent of current revenue)	26
Capital account restrictions	24
**Social globalization**	37
**(i) Data on personal contact**	33
Telephone traffic	25
Transfers (percent of GDP)	2
International tourism	26
Foreign population (percent of total population)	21
International letters (per capita)	25
**(ii) Data on information flows**	36
Internet users (per 1000 people)	37
Television (per 1000 people)	39
Trade in newspapers ((percent of GDP)	25
**(iii) Data on cultural proximity**	32
Number of McDonald’s restaurants (per capita)	47
Number of Ikea (per capita)	47
Trade in books (percent of GDP)	6
**Political globalization**	27
Embassies in country	25
Membership in international organizations	27
Participation in U.N. Security Council missions	22
International treaties	26

*Note* Weights may not sum to 100 because of rounding

## References

[CR1] Abotsi AK (2018). Influence of governance indicators on illicit financial outflow from developing countries. Contemporary Economics.

[CR2] Ades A, Di Tella R (1999). Rents, competition, and corruption. American Economic Review.

[CR3] Aguilera RV, Filatotchev I, Gospel H, Jackson G (2008). An organizational approach to comparative corporate governance: Costs, contingencies, and complementarities. Organization Science.

[CR4] Aidt T, Dutta J, Sena V (2008). Government regimes, corruption and growth: theory and evidence. Journal of Comparative Economics.

[CR5] Aïssaoui R, Fabian F (2015). The French paradox: Implications for variation in global convergence. Journal of International Management.

[CR6] Akça H, Ata AY, Karaca C (2012). Inflation and corruption relationship: Evidence from panel data in developed and developing countries. International Journal of Economics and Financial Issues.

[CR7] Akhter SH (2004). Is globalization what it’s cracked up to be? Economic freedom, corruption, and human development. Journal of World Business.

[CR8] Aligica PD, Tarko V (2014). Institutional resilience and economic systems: Lessons from Elinor Ostrom’s work. Comparative Economic Studies.

[CR9] Allison PD, Williams R, Moral-Benito E (2017). Maximum likelihood for cross-lagged panel models with fixed effects. Socius.

[CR10] Allred BB, Findley MG, Nielson D, Sharman JC (2017). Anonymous shell companies: A global audit study and field experiment in 176 countries. Journal of International Business Studies.

[CR11] Ang YY (2020). Unbundling corruption: Revisiting six questions on corruption. Global Perspectives.

[CR12] Aron J (2000). Growth and institutions: A review of the evidence. The World Bank Research Observer.

[CR13] Asongu S (2012). Globalization, (fighting) corruption and development: how are these phenomena linearly and nonlinearly related in wealth effects?. Journal of Economic Studies.

[CR14] Avnimelech G, Zelekha Y, Sharabi E (2014). The effect of corruption on entrepreneurship in developed vs non-developed countries. International Journal of Entrepreneurial Behaviour & Research.

[CR15] Bahoo S, Alon I, Paltrinieri A (2020). Corruption in international business: A review and research agenda. International Business Review.

[CR16] Bentler PM, Chou CP (1987). Practical issues in structural modeling. Sociological Methods & Research.

[CR17] Bhagwati J (2004). In defense of globalization.

[CR18] Brandl K, Darendeli I, Mudambi R (2019). Foreign actors and intellectual property protection regulations in developing countries. Journal of International Business Studies.

[CR19] Bryant CE, Javalgi RG (2016). Global economic integration in developing countries: The role of corruption and human capital investment. Journal of Business Ethics.

[CR20] Buckley PJ (2018). Towards a theoretically-based global foreign direct investment policy regime. Journal of International Business Policy.

[CR21] Cannizzaro AP (2020). Social influence and MNE strategic response to political risk: A global network approach. Journal of International Business Studies.

[CR22] Carr M, Chen MA (2001). Globalization and the informal economy: How global trade and investment impact on the working poor.

[CR23] Clegg J (2019). International business policy: What it is, and what it is not. Journal of International Business Policy.

[CR24] Cole DA, Maxwell SE (2003). Testing mediational models with longitudinal data: Questions and tips in the use of structural equation modeling. Journal of Abnormal Psychology.

[CR25] Country Watch. 2016. *United Arab Emirates: 2016 country review*. Retrieved from: http://www.countrywatch.com/content/pdfs/reviews/B46LL3ZL.01c.pdf. Accessed: December 18, 2020.

[CR26] Cuervo-Cazurra A (2016). Corruption in international business. Journal of World Business.

[CR27] Cuervo-Cazurra A (2018). Thanks but no thanks: State-owned multinationals from emerging markets and host-country policies. Journal of International Business Policy.

[CR28] Cuervo-Cazurra A, Gaur A, Singh D (2019). Pro-market institutions and global strategy: The pendulum of pro-market reforms and reversal. Journal of International Business Studies.

[CR29] Darendeli IS, Hill TL (2016). Uncovering the complex relationships between political risk and MNE firm legitimacy: Insights from Libya. Journal of International Business Studies.

[CR30] Das J, DiRienzo C (2009). The nonlinear impact of globalization on corruption. International Journal of Business and Finance Research.

[CR31] Di Pietra R, Melis A (2016). “Governance and corruption: Is history repeating itself?” Fostering a debate and inviting contributions from a multidisciplinary perspective. Journal of Management and Governance.

[CR32] Didzgalvyte-Bujauske M, Pereira ET, Osteikaite A (2019). The effect of globalization for economic growth of developing countries. Applied Economics: Systems Research.

[CR33] DiMaggio PJ, Powell WW (1983). The iron cage revisited: Institutional isomorphism and collective rationality in organizational fields. American Sociological Review.

[CR34] Dimant E, Tosato G (2018). Causes and effects of corruption: What has past decade’s empirical research taught us?. A survey. Journal of Economic Surveys.

[CR35] DiRienzo CE, Das J, Cort KT, Burbridge J (2007). Corruption and the role of information. Journal of International Business Studies.

[CR36] Donaldson L (2001). The contingency theory of organization.

[CR37] Doner RF, Schneider BR (2016). The middle-income trap: More politics than economics. World Politics.

[CR38] Dreher A (2006). Does globalization affect growth? Evidence from a new index of globalization. Applied Economics.

[CR39] Erez M, Gati E (2004). A dynamic, multi-level model of culture: From the micro-level of the individual towards the macro level of a global culture. Applied Psychology.

[CR40] Escresa I, Picci L (2016). Trends in corruptions around the world. European Journal on Criminal Policy and Research.

[CR41] Fanack.com. 2020. *Governance & politics of the UAE*. Retrieved from: https://fanack.com/united-arab-emirates/governance-and-politics-of-uae/#reform. Accessed: December 18, 2020.

[CR42] Faure GO, Fang T (2008). Changing Chinese values: Keeping up with paradoxes. International Business Review.

[CR43] Firebaugh G, Goesling B (2004). Accounting for the recent decline in global income inequality. American Journal of Sociology.

[CR44] Fiss PC, Hirsch PM (2005). The discourse of globalization: Framing and sensemaking of an emerging concept. American Sociological Review.

[CR45] Galang RMN (2012). Victim or victimizer: Firm responses to government corruption. Journal of Management Studies.

[CR46] Granovetter M (1985). Economic action and social structure: The problem of embeddedness. American Journal of Sociology.

[CR47] Granovetter M (1992). Economic institutions as social constructions: A framework for analysis. Acta Sociologica.

[CR48] Habib M, Zurawicki L (2002). Corruption and foreign direct investment. Journal of International Business Studies.

[CR49] Henisz W, Swaminathan A (2008). Introduction: Institutions and international business. Journal of International Business Studies.

[CR50] Hessels J, Terjesen S (2010). Resource dependency and institutional theory perspectives on direct and indirect export choices. Small Business Economics.

[CR51] Hoekman B, Nelson DR (2018). Reflecting on populism and the economics of globalization. Journal of International Business Policy.

[CR52] Holm H, Sorenson G (1995). Whose world order? Uneven globalization and the end of Cold War.

[CR53] Hu L, Bentler PM (1999). Cutoff criteria for fit indexes in covariance structure analysis: Conventional criteria versus new alternatives. Structural Equation Modeling: A Multidisciplinary Journal.

[CR54] Jensen NM, Li Q, Rahman A (2010). Understanding corruption and firm responses in cross-national firm-level surveys. Journal of International Business Studies.

[CR55] Jones A, Clark J (2013). A modern-day Icelandic saga”: Political places and negotiating spaces at the northern frontier of “Europe. European Urban and Regional Studies.

[CR56] Judge WQ, McNatt DB, Xu W (2011). The antecedents and effects of national corruption: A meta-analysis. Journal of World Business.

[CR57] Kar, D., & Spanjers, J. 2015. *Illicit financial flows from developing countries: 2004–2013*. Global Financial Integrity. Retrieved from: https://www.gfintegrity.org/wp-content/uploads/2015/12/IFF-Update_2015-Final-1.pdf. Accessed: December 18, 2020.

[CR58] Kaufmann, D. 2005. *Myths and realities of governance and corruption*. Natural Resource Governance Institute; The Brookings Institution. Retrieved from: https://papers.ssrn.com/sol3/papers.cfm?abstract_id=829244. Accessed: December 18, 2020.

[CR59] Kautto D (2018). Social influences in cross-border entrepreneurial migration policy. Journal of International Business Policy.

[CR60] Kenny, D. A. 2020. *Measuring model fit*. Retrieved from: http://davidakenny.net/cm/fit.htm. Accessed: December 18, 2020.

[CR61] Kim H, Kim H, Hoskisson RE (2010). Does market-oriented institutional change in an emerging economy make business-group-affiliated multinationals perform better? An institution-based view. Journal of International Business Studies.

[CR62] Kline RB (1998). Software programs for structural equation modeling: Amos, EQS, and LISREL. Journal of Psychoeducational Assessment.

[CR63] KOF. 2013. KOF Index of Globalization 2013. Retrieved from: http://globalization.kof.ethz.ch/media/filer_public/2013/03/25/method_2013.pdf. Accessed: December 18, 2020.

[CR64] KOF. 2017. *KOF index of globalization*. Retrieved from: http://globalization.kof.ethz.ch/. Accessed: December 18, 2020.

[CR65] Koning M, Mertens G, Roosenboom P (2018). Drivers of institutional change around the world: The case of IFRS. Journal of International Business Studies.

[CR66] Krammer SMS (2017). Greasing the wheels of change: Bribery, institutions, and new product introductions in emerging markets. Journal of Management.

[CR67] Krugman P (2008). Trade and wages, reconsidered. Brookings Papers on Economic Activity.

[CR68] Kushida KE, Shimizu K, Oi JC (2013). Syncretism: The politics of economic restructuring and system reform in Japan.

[CR69] Kwok CC, Tadesse S (2006). The MNC as an agent of change for host-country institutions: FDI and corruption. Journal of International Business Studies.

[CR70] Lalountas DA, Manolas GA, Vavouras IS (2011). Corruption, globalization and development: How are these three phenomena related?. Journal of Policy Modeling.

[CR71] Lang J, Bliese PD, Lang JWB, Adler AB (2011). Work gets unfair for the depressed: cross-lagged relations between organizational justice perceptions and depressive symptoms. Journal of Applied Psychology.

[CR72] Lawrence TB, Suddaby R, Leca B (2009). Institutional work: Actors and agency in institutional studies of organizations.

[CR73] Levie J, Autio E (2011). Regulatory burden, rule of law, and entry of strategic entrepreneurs: An international panel study. Journal of Management Studies.

[CR74] Little TD, Preacher KJ, Selig JP, Card NA (2007). New developments in latent variable panel analyses of longitudinal data. International Journal of Behavioral Development.

[CR75] Lorenzen M, Mudambi R, Schotter A (2020). International connectedness and local disconnectedness: MNE strategy, city-regions and disruption. Journal of International Business Studies.

[CR76] MacCallum RC, Austin JT (2000). Applications of structural equation modeling in psychological research. Annual Review of Psychology.

[CR77] MacCallum RC, Browne MW, Sugawara HM (1996). Power analysis and determination of sample size for covariance structure modeling. Psychological Methods.

[CR78] Marano V, Tashman P, Kostova T (2017). Escaping the iron cage: Liabilities of origin and CSR reporting of emerging market multinational enterprises. Journal of International Business Studies.

[CR79] Mauro P (1995). Corruption and growth. Quarterly Journal of Economics.

[CR80] Medina LF, Bucheli M, Kim M (2019). Good friends in high places: Politico-economic determinants of the expropriation and taxation of multinational firms. Journal of International Business Policy.

[CR81] Méon PG, Sekkat K (2005). Does corruption grease or sand the wheels of growth?. Public Choice.

[CR82] Meyer JW, Boli J, Thomas GM, Ramirez FO (1997). World society and the nation-state. American Journal of Sociology.

[CR83] Meyer KE, Li C, Schotter APJ (2020). Managing the MNE subsidiary: Advancing a multi-level and dynamic research agenda. Journal of International Business Studies.

[CR84] Meyer JW, Rowan B (1977). Institutionalized organizations: Formal structures as myth and ceremony. American Journal of Sociology.

[CR85] Nebus J (2019). Will tax reforms alone solve the tax avoidance and tax haven problems?. Journal of International Business Policy.

[CR86] North D (1990). Institutions, institutional change and economic performance.

[CR87] Nye JS (1967). Corruption and political development: A cost-benefit analysis. American Political Science Review.

[CR88] Olabisi M (2019). Bridging the enforcement gap in international trade: Participation in the New York Convention on arbitration. Journal of International Business Policy.

[CR89] Ozawa T (2019). A note on Dani Rodrik, “Populism and the economics of globalization**”**. Journal of International Business Policy.

[CR90] Peng MW, Ahlstrom D, Carraher SM, Shi W (2017). An institution-based view of global IPR history. Journal of International Business Studies.

[CR91] Petricevic O, Teece DJ (2019). The structural reshaping of globalization: Implications for strategic sectors, profiting from innovation, and the multinational enterprise. Journal of International Business Studies.

[CR92] Pfarrer MD, Smith KG, Bartol KM, Khanin DM, Zhang X (2008). Coming forward: The effects of social and regulatory forces on the voluntary restatement of earnings subsequent to wrongdoing. Organization Science.

[CR93] Pfeffer J, Salancik G (1978). The external control of organizations: A resource dependence perspective.

[CR94] Potrafke N (2015). The evidence on globalization. World Economy.

[CR95] Rodrik D (1982). Managing resource dependency: The United States and Japan in the markets for copper, iron ore and bauxite. World Development.

[CR96] Rodrik D (2011). The globalization paradox: Democracy and the future of the world economy.

[CR97] Rodrik D (2018). Populism and the economics of globalization. Journal of International Business Policy.

[CR98] Rose-Ackerman S (1978). Corruption: A study of political economy.

[CR99] Scott WR (2001). Institutions and organizations.

[CR100] Sharman JC (2017). Illicit global wealth chains after the financial crisis: Micro-states and an unusual suspect. Review of International Political Economy.

[CR101] Sherer PD, Lee K (2002). institutional change in large law firms: A resource dependency and institutional perspective. Academy of Management Journal.

[CR102] Stiglitz JE (2003). Globalization and growth in emerging markets and the new economy. Journal of Policy Making.

[CR103] Tihanyi L, Hegarty WH (2007). Political interests and the emergence of commercial banking in transition economies. Journal of Management Studies.

[CR104] Transparency International. 2017a. *What is corruption?* Retrieved from: https://www.transparency.org/what-is-corruption/. Accessed: December 18, 2020.

[CR105] Transparency International. 2017b. *Corruption perception index*. Retrieved from: https://www.transparency.org/en/cpi. Accessed: December 18, 2020.

[CR106] Ugur M (2014). Corruption’s direct effects on per-capita income growth: A meta-analysis. Journal of Economic Surveys.

[CR107] Voyer PA, Beamish PW (2004). The effect of corruption on Japanese foreign direct investment. Journal of Business Ethics.

[CR108] Wang SL, Li D (2019). Responding to public disclosure of corporate social irresponsibility in host countries: Information control and ownership control. Journal of International Business Studies.

[CR109] Westaway J (2012). Globalization, sovereignty and social unrest. Journal of Politics and Law.

[CR110] Westland JC (2010). Lower bounds on sample size in structural equation modeling. Electronic Commerce Research and Applications.

[CR111] Wilson JD (2011). Resource nationalism or resource liberalism: Explaining Australia’s approach to Chinese investment in its mineral sector. Australian Journal of International Affairs.

[CR112] Witt MA (2019). De-globalization: Theories, predictions, and opportunities for international business research. Journal of International Business Studies.

[CR113] World Bank. 2017. *GDP per capita (current US$)*. Retrieved from: https://data.worldbank.org/indicator/NY.GDP.PCAP.CD. Accessed: December 18, 2020.

[CR114] WTO. 2016. *Trade in 2016 to grow at slowest pace since the financial crisis*. Retrieved from: https://www.wto.org/english/news_e/pres16_e/pr779_e.htm. Accessed: December 18, 2020.

[CR115] Yenkey CB (2015). Mobilizing a market: Ethnic segmentation and investor recruitment into the Nairobi Securities Exchange. Administrative Science Quarterly.

[CR116] Yi J, Meng S, Macaulay CD, Peng MW (2019). Corruption and foreign direction investment phases: The moderating role of institutions. Journal of International Business Policy.

[CR117] Yuchtman-Yaar E, Inbar M (1986). Social distance in the Israeli-Arab conflict: A resource-dependency analysis. Comparative Political Studies.

[CR118] Zenger TR, Lazzarini SG, Poppo L, Ingram P, Silverman B (2001). Informal and formal organization in new institutional economics. The new institutionalism in strategic management.

[CR119] Zimmerman MA, Zeitz GJ (2002). Beyond survival: Achieving new venture growth by building legitimacy. Academy of Management Review.

